# An Overview of Nanotechnologies for Drug Delivery to the Brain

**DOI:** 10.3390/pharmaceutics14020224

**Published:** 2022-01-19

**Authors:** Ahsan Ayub, Shawn Wettig

**Affiliations:** School of Pharmacy, University of Waterloo, 10 Victoria St S A, Kitchener, ON N2G 1C5, Canada; ahsan.ayub@uwaterloo.ca

**Keywords:** nanoparticle, nanomedicine, drug delivery, brain, cancer, brain tumours, polymer, targeting, micelle, dendrimer

## Abstract

Drug delivery to the brain has been one of the toughest challenges researchers have faced to develop effective treatments for brain diseases. Owing to the blood–brain barrier (BBB), only a small portion of administered drug can reach the brain. A consequence of that is the need to administer a higher dose of the drug, which, expectedly, leads to a variety of unwanted side effects. Research in a variety of different fields has been underway for the past couple of decades to address this very serious and frequently lethal problem. One area of research that has produced optimistic results in recent years is nanomedicine. Nanomedicine is the science birthed by fusing the fields of nanotechnology, chemistry and medicine into one. Many different types of nanomedicine-based drug-delivery systems are currently being studied for the sole purpose of improved drug delivery to the brain. This review puts together and briefly summarizes some of the major breakthroughs in this crusade. Inorganic nanoparticle-based drug-delivery systems, such as gold nanoparticles and magnetic nanoparticles, are discussed, as well as some organic nanoparticulate systems. Amongst the organic drug-delivery nanosystems, polymeric micelles and dendrimers are discussed briefly and solid polymeric nanoparticles are explored in detail.

## 1. Introduction and Background

Naturally occurring products have been used to remedy various diseases and illnesses throughout history [1]. The aggressive growth of biomedical research in recent decades has led to an exponential increase in population. Ironically, this rise in population has become an unprecedented source of strain on the healthcare industry globally, leading to an increase in costs and a shortage in personnel [2,3]. Over the last few decades, improved therapeutic modalities have begun to surface as potential solutions to this burden on healthcare. Two of the major directions researchers have steered towards for the creation of such improved therapeutic modalities are the fields of *drug discovery* and *drug delivery*.

Drug discovery uses the principles of organic chemistry, biochemistry, biology and pharmacology to create novel synthetic or semi-synthetic drugs [4]. The practice of discovering new drugs has likely been around since the very early days of civilization, with the oldest records suggesting a concoction of various herbs, shrubs, leaves, minerals and animal excreta, being used for medicinal purposes in ancient Egypt around 1500 BC [5]. However, the true potential of drug discovery has only become apparent in the last couple of centuries, as scientists have been able to use the technological advancements in chemistry and biology to truly define how crucially important the drug discovery process can be [4]. Having said that, whilst effective (and necessary), the modern drug development and commercialization process is not without pitfalls. The process is expensive, lengthy and labour-intensive [6,7]. Thankfully, using drug-delivery systems (DDSs) to more efficiently deliver existing drugs to target tissues can mitigate those issues.

In the most basic terms, DDSs refer to techniques used to administer medications with enhanced safety and efficacy by regulating their rate, time and location of release in the body [8]. Conventional drug-administration techniques are still widely used, but they present with certain limitations. Novel drug-delivery systems can be used to offset a number of such limitations. Drug administration can either be local or systemic, depending on the disease and requirements using novel drug-delivery systems. Recently, the delivery of drugs orally has been at the forefront of research in the field of drug delivery due to the ease and convenience of administration. This is especially true for treatments of chronic conditions involving continuous medication for extended periods of time. One of the major challenges for oral administration of conventional drugs is the harsh acidic conditions of the gastrointestinal tract and first-pass metabolism. These issues could theoretically be resolved using pH-resistant drug-delivery systems able to carry the drug and releasing only once it has reached the systemic bloodstream [8,9].

Nano-drug-delivery systems are DDSs operating at the nano-scale and offer significant benefits. Nano-delivery is an important part of the still-evolving field of nanomedicine. Nanomedicine is a multidisciplinary field comprising of aspects of nanotechnology, chemistry, biochemistry and pharmaceutical sciences. Nanotechnology can be described as the science of synthesis, characterization and application of materials and devices with at least one dimension falling in the “nano” scale [10,11]. Nano-delivery systems offer certain advantages over traditional drug-delivery methods. For instance, they may be able to deliver drugs to treat a number of diseases and target a variety of tissues within the body with high specificity. Targeted therapy using nano-delivery systems could also lead to an indirect reduction in the side effects accompanied by several drugs, since they should, in theory, prevent or significantly reduce drug interactions with non-target tissues. A direct consequence of the reduction in unwanted drug interactions would lead to a lower dosage of the drug being required to treat the condition, ultimately leading to an overall decrease in costs [12,13,14].

## 2. Delivery of Therapeutics to the Brain

### 2.1. The Blood–Brain Barrier (BBB)

The brain is the most important organ in the human body; it is what enables us to do everything we do, from tying our shoelaces to solving differential equations. Therefore, it is crucial that no harm comes to it. Through the years, to ensure its safety, humans have evolved several physiological and biochemical defence systems that protect the brain. One of the most important of those defence systems is the blood–brain barrier (BBB)—a highly selective, partially permeable barrier between the brain and the rest of the body. The BBB is what allows specific molecules from the general circulatory system to reach the brain and the central nervous system (CNS) [15,16].

The blood–brain barrier is highly selective in what it perceives to be worthy of reaching the brain. Its very strict regulation of solutes, ions and molecules into the CNS and brain is a result of the tight junctions formed by densely packed endothelial cells [16]. Apart from the physical barrier that the tight junctions of the endothelial cells provide, the BBB also has a complex efflux transporter system which actively removes molecules from the brain and the cerebrospinal fluid transporting them back the into systemic circulation [17]. Such high regulation of molecules to reach the CNS by crossing the BBB has posed a big challenge for scientists to treat CNS or brain disorders or diseases, as many of the drugs that need to reach the brain to be effective are unable to do so because of the BBB’s highly selective nature [18].

There are several physical and chemical characteristics molecules must possess to be able not only to pass through the barrier, but to reach their target cells or tissue. The brain’s interstitial fluid is an aqueous environment, which means that any molecule that is able to cross the BBB needs to be at least somewhat hydrophilic to make it to the brain [19]. Though a higher degree of lipophilicity may aid the molecule to permeate through the BBB, it may also end up causing less of that molecule to reach the target area. Therefore, a moderately lipophilic molecule or compound has the best overall chance of permeating through the BBB and reaching the brain [20]. The weight of the molecule also affects its chances of permeating through the BBB. Generally, lighter molecules have a higher chance of passing through the barrier and reaching the brain; however, the upper bound of molecular weights of the compounds depends on the mode of transportation [19,20].

#### Transport of Molecules across the BBB

**Figure 1:**
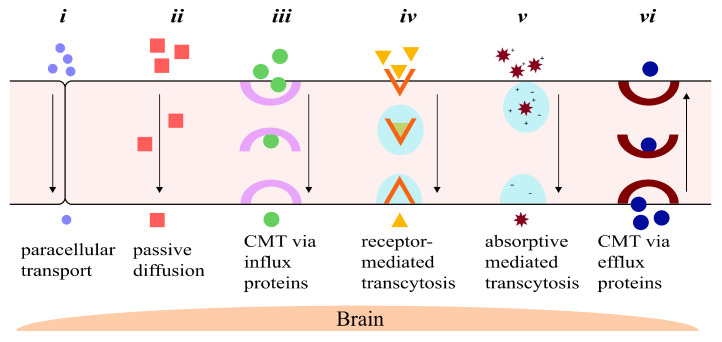
A schematic representation of the six major ways drugs and other molecules travel across the blood–brain barrier: (**i**) paracellular transport between the tight junctions between the endothelial cells by small, hydrophilic molecules; (**ii**) passive diffusion through the endothelial cells of small lipophilic drugs; (**iii**) carrier-mediated transport of larger lipophilic molecules by influx carrier proteins; (**iv**) receptor-mediated transcytosis of larger molecules with affinity towards specific receptors; (**v**) absorptive-mediated transcytosis of large, cationic molecules by binding and ultimately absorbing into the luminal surface of the endothelial cell; and (**vi**) transport of unwanted molecules back into systemic circulation by efflux transporters.

Foreign molecules can pass through the BBB either via paracellular (between the cells), or transcellular (through the cells, also known as transcytosis) transport systems. There are six main ways via which molecules can transport across the BBB, as follows, summarized in Figure 1:

- Paracellular transport: The transport of molecules through the intercellular spaces between the endothelial cells is the most restrictive form of transport through the BBB due to the tight junctions (Figure 1i) [21]. Paracellular transport through the BBB is mostly dictated by the environmental concentration gradient; i.e., a greater concentration gradient leads to more transport between the endothelial cells, with minor contributions from the molecule’s surface charge, its size and its lipophilicity [19,21]. The tight junctions contain tiny aqueous pores, which means only small hydrophilic compounds are able to make it across the BBB [21,22].
- Diffusion: Passive transcellular diffusion across the BBB through the endothelial cells (Figure 1ii) mainly depends on the lipophilicity of the molecule; higher lipophilicity means higher diffusion. Size also plays an important role here, with smaller compounds being relatively easily permeated. As with paracellular diffusion of molecules between the endothelial cells, the environmental concentration gradient also affects this pathway [19,22]. Compounds and particles that cross the BBB through the lipid-mediated transcellular diffusion must have a molecular weight of less than 500 Da [23,24]. A 100-fold decrease in BBB permeability has been reported for molecules of molecular weight of 450 Da when compared to smaller, 200 Da molecules [24,25].
- Influx transporters: For molecules that are unable to cross the BBB via diffusion due to size or molecular weight restrictions, a very efficient protein-transporter system is in place to help them across. Transport across the BBB through influx transporter proteins, also referred to as carrier-mediated transport (CMT), involves transporters binding to and carrying specific molecules through the BBB (Figure 1iii) [26]. These transporters assist slightly larger lipophilic molecules to permeate through the BBB that show an affinity towards specific endogenous BBB transporters. Molecules are bound to the transporter and carried across the endothelial cell lining of the BBB into the CNS [18,26].
- Receptor-mediated transcytosis (RMT): One of the important ways macromolecules pass through the BBB is via RMT. Receptors selective towards specific ligands enable a number of large molecules such as sugars, proteins, hormones, etc., to pass through the BBB [27]. RMT takes place in the following three distinct steps: Refs. [27,28,29]
  (a) Receptor-mediated endocytosis of molecule by the receptor;
  (b) Transport of vesicle across the membrane;
  (c) Release of molecule into the extracellular space of endothelial cells.
  RMT differs from CMT mechanistically. Protein carriers that are able to freely move across the endothelial cells of the BBB aid a substrate by binding to and carrying it across the BBB themselves. In contrast, molecules crossing the BBB using RMT only bind to specific receptor-proteins on the membrane of the endothelial cells to transcytose across the BBB (Figure 1iv) [18,30].
- Absorptive-mediated transcytosis (AMT): Cationic molecules are not readily transported across the BBB via the previously discussed pathways; however, they have the ability to bind to and absorb into the luminal surface of the endothelial cells that make up the BBB [31]. Following the bond between the endothelial cells and the molecule, AMT follows the same path as RMT, in which endocytosis is followed by transport across the endothelial cell lining, ending with exocytosis of the molecule (Figure 1v) [28,31].
- Efflux transporters: This transport system is in charge of discarding unwanted molecules back into the systemic bloodstream using efflux proteins via CMT (Figure 1vi). The efflux transporter system employed by the BBB is very effective and is one of the major hurdles for drugs to treat brain or CNS diseases [28,32]. One of the most important proteins in the efflux pump is the P-glycoprotein (P-gp), which is responsible for the elimination of a number of drugs and molecules from the BBB [33].

### 2.2. Drug-Delivery Routes to the Brain

In total, 98% of all small drugs (<500 Da) and 100% of all large drugs (>500 Da) do not cross the BBB and more than 95% of all small drugs that make it across are inactive in the CNS [26,27]. For a molecule, such as a drug, to pass through the BBB and reach its target cells, it has to fulfill a number of physico-chemical requirements. In order for the drug to permeate through the BBB via paracellular transport, it needs (1) to be small enough to pass through the intercellular space of the endothelial cells, (2) to be hydrophilic enough to dissolve into the aqueous tight junction pores, (3) not to be a substrate for a number of the efflux transporters such as P-glycoprotein (P-gp) found past the BBB and (4) not to be a substrate for the several enzymes that are in the BBB whose only purpose is to break down unwanted molecules before reaching the brain [19,34].

If the intention is for the drug to pass through the BBB via transcellular diffusion, it needs to be small and lipophilic enough to pass through the endothelial cell lining, but also needs to possess some degree of hydrophilicity for it to be able to partition into the aqueous environment of the brain’s interstitial fluid [31,35]. For transport through the BBB via RMT, AMT, or CMT, efflux pumps, ligand-specific receptors and enzymes need to be considered, as these are all in place to protect the brain against unwanted foreign chemicals [19,30,35,36]. Largely because of the BBB, delivering drugs and other therapeutics to the brain or the CNS has been a major hurdle for treating brain and CNS diseases and disorders. Due to the fact that a significant fraction of the administered dose of most drugs is unable to cross the BBB, the amount of drug administered needs to be very high to be efficacious [37]. Therefore, several strategies have been employed to develop novel systems for improved delivery of drugs past the BBB to reach the brain [38]. Some of the common modes of delivering drugs to the brain or the CNS are described below:

- *Viral vectors:* As part of their replication cycle, viruses attack their host to introduce their own genetic material into the host cell. The inserted genetic material is composed of basic instructions on how to produce more viruses. The viruses end up effectively taking over the host cell completely to fulfill their own needs [39]. The infected host cells carry out these instructions, with more and more viruses being produced, eventually cascading into taking over more host cells [39,40]. The genetic material that guides the host cells to assist the replication of viruses can be substituted with genetic instructions that would be beneficial for the host, for example, instructions to invade, infect and kill cancerous cells. Essentially, viral vectors can be used to deliver specific genes to fight or prevent diseases (gene therapy) [41,42]. In addition to gene therapy, viral vectors have been generating interest as drug carriers in recent years. The drugs can be encapsulated or infused with the vector, which can be functionalized for targeted delivery. Overall, there are still several issues associated with using viral vectors as drug-delivery systems, mainly linked with their high cost of production and their safety, as administration of viruses always carries a certain level of risk [43,44,45].
- *Exosomes:* Exosomes are naturally occurring vesicles that are being used as drug carriers to penetrate the BBB for smaller drugs, proteins and nucleic acids; they are of increasing interest due to their high biocompatibility. Functionalized exosomes have been seen to be able to cross the BBB through RMT [46]. The limitations associated with using exosomes as drug-delivery systems have to do with the variety of drugs that can be loaded into them. Whether a drug is a suitable contender to be used in an exosome DDS is mainly dependant on the drug’s physical and chemical characteristics [43,47].
- *Drug delivery via active transporters:* Essential amino acids, such as phenylalanine, leucine, tyrosine, isoleucine, valine, tryptophan, methionine and histidine, travel across the BBB into the brain and CNS via carrier-mediated influx. Smaller drugs with appropriate physico-chemical properties have been linked with these amino acids that work as active transporters and carry the molecule through the BBB endothelial cells. Similar to exosomes, the properties of the drug dictate whether using amino acids as active transporters for the delivery of the drug to the brain is going to be successful [48].
- *Enhancing paracellular transport:* The BBB has been shown to be disruptable, enabling certain drugs to pass through via paracellular diffusion which otherwise would not be able to [28,49]. There are two main ways whereby the BBB can be disrupted, i.e., by using osmotic agents that draw the cellular water out of the endothelial cells, resulting in them shrinking, enabling the drug passage through the tight junctions; and by using chemical agents that generate a temporary inflammatory reaction in the endothelial cells, which results in the tight junctions loosening temporarily, allowing the drug to pass through [38,49]. Paracellular transport is restrictive, which means only small hydrophilic drugs can cross through a disrupted BBB [38,50].
- *Modification of drugs for transcytosis:* Drugs can be modified by changing their physico-chemical properties (lipophilicity, size, charge, shape, etc.) to enhance AMT, or by conjugating them with specific ligands or antibodies to trigger RMT [38]. This is a very promising strategy for drug delivery to the brain owing to its range of versatility and cost-effectiveness relatively to other delivery systems [34,38,50].
- *Nanotechnology:* Nanotechnology-based drug-delivery systems are being researched for enhanced delivery of drugs to the brain and the CNS for a variety of reasons [50]. Specific examples are explored in Section 3 and Section 4.

### 2.3. Glioblastomas

Glioblastomas are Grade IV astrocytomas and are the commonest and most aggressive of all primary brain tumours with a global incidence rate of 3.19 per 100,000 according to the World Health Organization (WHO) [51,52]. They account for 12–15% of all malignant intracranial and 50–60% of all astrocytic tumours [52]. Glioblastomas are also commonly referred to as “*Glioblastoma Multiforme*” (GBM) and are considered the deadliest type of primary brain tumours due to their rapid and aggressive growth. Less than 20% of the patients diagnosed with GBM survive for longer than 2 years and less than 5% live past 5 years [52,53].

#### 2.3.1. Treatment of GBMs

There are numerous challenges when treating GBMs. The tumour cells often develop resistance to conventional therapy due to the high tumour heterogeneity. Heterogeneity refers to the morphological and phenotypic differences between the tumour cells. A high degree of heterogeneity leads to cells behaving differently from each other, making the therapeutic agents’ effectiveness vary considerably. Such differences between GBM cells is also a root of notable challenges in the diagnosis of the cancer [54]. The high selectivity of the blood–brain barrier is another major challenge for GBM treatment, as most conventional drugs are not able to reach the brain. Due to the aggressive growth and heterogeneity among GBM tumour cells, a multidisciplinary approach is taken for its treatment. The first step is the surgical removal of tumour, followed by simultaneous radiation therapy and chemotherapy, all of which see from very low to low success rates in most cases [55].

#### 2.3.2. GBMs and Transferrin

Transferrins are a family of glycoproteins whose main biological function is thought to be related to their ability to bind with iron. At least three different types of glycoproteins in the transferrin family have been recognized, each thought to serve a unique function; those are: serum-Transferrin (Tf), ovo-Transferrin (oTf) and Lactoferrin (Lf) [56]. Lactoferrin is named as such due to it being predominantly found in mammalian milk [57]. Ovo-transferrin is predominantly found in avian egg-whites [58]. Serum transferrin (referred to simply as transferrin or Tf for the remainder of the paper) is responsible for the systemic transport of iron (and other metal ions) from intake sites to the general circulation. Apart from transport of iron, Lf and oTf are thought to utilize their iron-binding properties to act as antimicrobials by snatching (chelating to) the iron, which plays an important role in microbial activity [56,59].

Tf selectively binds to the transferrin receptor (TfR) [60]. A significant amount of TfR is present on the brain capillary endothelial cells and is responsible for transporting iron to the brain through RMT of iron-bound Tf [60,61,62]. In normal brain tissue, the highest levels of TfR have been seen to be on the medulla oblongata and the hippocampus, while TfR levels are noticeably lower in the cortex, thalamus and cerebellum [52]. While no brain tumours have presented with a statistically elevated expression of TfR in the cortex, linings of astrocytoma cells, including GBMs’, have been observed to have much higher levels of TfR [53].

## 3. Characteristics of an Effective Nanoparticle DDS

### 3.1. Biocompatibility

Undoubtedly, the most important factor when designing a nanoparticulate (or otherwise) drug-delivery system is its safety. A nanoparticle (NP)’s interactions with living cells, tissues and organs is crucial to consider and the nanoparticle and all its components must be safe and non-toxic, i.e., they must be biocompatible [63,64,65]. Another important parameter a nano-delivery system must possess is stability in physiological environments. How long after administration the NP starts to degrade and how well its metabolites behave with living cells and tissues is indicative of how effective the drug-delivery system is going to be [65]. The immune system jumps into action as soon as a foreign molecule enters the body. An ideal drug-carrier must be able to withstand the attack by the immune system and retain its shape and structural integrity for the intended period of time [13]. Several factors impact the biocompatibility and toxicity profiles of nanoparticulate drug-delivery systems. One major reason why so many different types of functionalization techniques are being investigated so thoroughly is to minimize the harmful properties of a drug-delivery system [63,64,66]. In addition to biocompatibility, a drug-carrier that biodegrades must break down into non-toxic and easy-to-eliminate metabolites or one that can be eliminated by the body completely. However, there are some polymers such as poloxamers that are of special interest owing to their lack of biodegradability [65]. Other factors that are extremely important when considering a nanoparticle drug-delivery system is cellular internalization or uptake, both of which are properties that can be influenced by any of the other characteristics of a DDS described below [66,67].

### 3.2. Size, Surface Morphology and Functionalizability

After biocompatibility, perhaps, the most important factor influencing the choice of the type of drug-delivery system is the size of the resulting nanoparticles. The size of the nanoparticle plays a crucial role in determining its effectiveness as a DDS and has been shown to be a major factor in determining its pharmacology, cellular uptake and targeting ability [68]. Smaller particles have demonstrated the ability to pass through cellular membranes with relative ease (to a varying degree), compared to larger ones [68]. The release of the drug is also highly influenced by the particle size, with smaller particles generally showing quicker release [69,70]. Finally, a given nano-delivery system must be one that can be functionalized in a variety of ways to modify its physico-chemical and pharmacological properties [63,71].

### 3.3. Surface Charge

The surface charge of a nanoparticulate DDS is another major factor that determines the cell internalization profile of the drug-delivery system. Positively charged NPs generally have higher uptake into cells than negatively charged ones [68,72]. The charge of the nanoparticulate surface also affects the circulation times, elimination processes and targeting abilities. For example, a highly positively charged NP is more prone to adsorbing plasma and surface proteins, which, ultimately, leads to aggregation and elimination from the body without it serving its full purpose [73,74].

### 3.4. Drug Loading Capacity

The amount of drug that can be loaded onto the delivery system is also an important consideration. Drugs can be loaded into/onto different NPs using a number of techniques, each of which provides its own benefits and drawbacks. The physical characteristics of the drug are also key factors here, as they impact the drug loading. Appropriate nanoparticulate DDSs should have a high drug loading capacity, irrespective of the technique used for loading [70,75].

### 3.5. Functionalizability

Nanotechnology has provided attractive and versatile modes of unconventional drug-delivery systems, a major reason of which is the high degree of functionalization that can be performed to obtain drug carriers with the desired physico-chemical properties [69,76]. While there are multiple strategies that are used for the functionalization of nanoparticulate DDSs, some techniques that are more commonly utilized to achieve specific properties in the resulting nanoparticle are discussed in the following subsections.

#### 3.5.1. End-Group Modification

End-group modification is one method that can be utilized for functionalization. Depending on what physico-chemical properties are required, a variety of different functional groups can be introduced onto the nanoparticles through covalent end-group modification [77]. The functionalization of nanoparticles through end-group modifications can be used to manipulate the NPs’ net charge, tertiary structure, solubility (lipophilicity), targeting ability and their stability in physiological environments [63,77].

#### 3.5.2. Crosslinkage

Two or more molecules can be joined together through covalent bonds; this joining is referred to as chemical crosslinking. Such covalent bonds can be made with the use of specific types of chemicals, referred to as ‘crosslinkers’ [78]. These come in a variety of shapes and sizes, each with different physico-chemical properties and each creating the crosslinkage using different mechanisms. Crosslinkers possess reactive ends capable of covalently binding to specific functional groups on a target molecule [78]. Crosslinkage of biological molecules such as proteins, peptides, antibodies, sugars, etc., is appropriately termed bioconjugation. Crosslinker chemistry can be used for bioconjugation of many biomolecules onto other molecules. Bioconjugation to polymers has been a tempting application of crosslinkers in drug delivery due to the versatility in the ways they can be used for a wide variety of biomolecules [79]. There are four main types of crosslinkers, three of which are discussed.

Two of the most common crosslinkers used in nanomedicine for bioconjugation are the homobifunctional and heterobifunctional crosslinkers [80]. Homobifunctional crosslinking agents contain the same functional group as their target molecule and are normally used in simple one-step reactions. Monomers can be polymerized using these crosslinkers as these are capable of linking similar functional groups to each other. Identical functional groups to the target molecule on homobifunctional crosslinking agents lead to poor reactivity, making them unsuitable to carry out bioconjugation [81]. In addition, these crosslinking agents pose several issues, such as formation of poorly defined conjugates, excessive unwanted crosslinkage and overall instability during reactions [80,82]. Heterobifunctional crosslinkers, in comparison, have different reactive ends from the target molecule, making them more selective than homobifunctional crosslinkers. These normally present with more stability and control over unwanted crosslinkages between functional groups; therefore, they are favoured over homobifunctional crosslinkers for bioconjugation purposes [80,81,83].

The size of a nanoparticle is a major consideration when being used for drug-delivery purposes. As such, maximum control over the length of a polymer to be used as a drug-delivery system is critical. This is where a third class of crosslinkers, zero-length crosslinkers, comes in. These function similarly to the previously discussed crosslinkers, but with one major difference [84]. Where bioconjugation through homo- and heterobifunctional crosslinkers could have variable spacing between the biomolecule and the polymer depending on the length of the crosslinker, bioconjugation through zero-length crosslinkers ensures minimal distance between the bioconjugate and the polymer. These are the smallest molecules that can be used for crosslinking and the bond formed from bioconjugation through these contains no additional atoms. With zero-length crosslinking agents, an atom of the biomolecule is directly attached to the polymer without any intervening linker or space [84,85].

Carbodiimides are among the most widely used and reliable class of zero-length crosslinkers. Carbodiimide linkers covalently bridge primary amines (RNH${}_{2}$) to carboxylic acids [R(C=O)OH] to form amide [R(C=O)NR${}^{\prime }$] functional groups. Not only can they crosslink peptides and proteins to polymers and other molecules, they can also be used to bridge two proteins, a peptide and a protein, a carbohydrate and a protein and any combination thereof [86]. *N-Ethyl-N${}^{\prime }$-(3-dimethylaminopropyl)carbodiimide hydrochloride* (EDC) is the most popular crosslinker used in bioconjugation reactions. EDC’s water solubility is one of its key features in bioconjugation applications as most biomacromolecules are soluble in aqueous buffer solutions [85].

**Figure 2:**
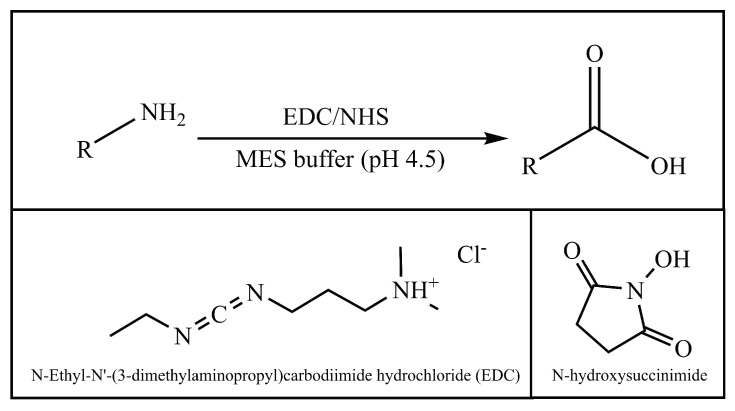
A generic reaction scheme showing the conversion of terminal amino groups into carboxylic acid groups that be used for functionalization using EDC and NHS.

One of the most dependable methods of conjugating peptides and proteins with polymeric nanoparticles containing terminal carboxyl (COOH-) groups without significantly affecting its size is using zero-length carbodiimide crosslinkers. Amide formation between such molecules using EDC and *N-hydroxysuccinimide* (NHS) has been successfully shown in several published studies. The generic reaction scheme to conjugate peptides using NHS and EDC can be seen in Figure 2 [84,85,87].

**Figure 3:**
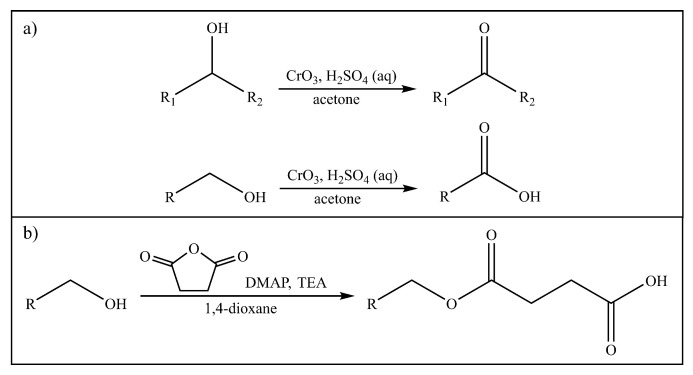
Generic reaction schemes that can be used to convert primary hydroxyl (-OH) groups into functionalizable carboxylic groups using (**a**) Jones oxidation and (**b**) esterification using an acid anhydride (succinic anhydride, in this example).

Transferrin is a glycoprotein that has free primary amino groups. Polymers that have available hydroxyl functional groups and no carboxyl groups are unsuited for carbodiimide crosslinkage; however, they can be “activated” through end-group modifications. Their terminal alcohols can be oxidized into carboxyl acid groups using oxidizers such as Jones reagent to obtain a carboxyl-terminated polymer [88,89]. Jones oxidation is a technique used to oxidize primary and secondary alcohols into carboxylic acids and ketones, respectively, commonly (Figure 3a). Jones reagent is prepared by the dissolution of chromium trioxide (CrO${}_{3}$) in aqueous sulphuric acid [90,91]. Another way this can be achieved is through esterification using an acid anhydride (for example, maleic anhydride or succinic anhydride). A generic reaction scheme can be seen in Figure 3b [89,92,93].

Following the activation, such copolymers are able to link to proteins such as Tf or bovine serum albumin (BSA) using EDC and NHS [94]. The crosslinking of Tf onto various different types of nanoparticles has shown to be an effective functionalization technique in a number of published studies [95,96,97,98,99].

NPs can also be linked to functional groups that do not affect their physico-chemical characteristics significantly, but rather change their biochemical ones; an example of such a modification would be linking a moiety to manipulate the rate of the NPs’ degradation after administration. Nanoparticles can be conjugated with ligands using crosslinkers that present affinity towards a specific receptor [100]. For example, an overexpression of folate receptor alpha (FRα) has been seen in some ovarian, lung and breast cancer cells; this can be exploited by functionalizing a drug carrier with folate, which binds specifically to FRα, for active drug targeting [101]. Due to foreign molecules’ inability to easily permeate through the BBB, nanoparticulate DDSs can be very useful for targeted delivery of drugs to the brain. Numerous biomolecule ligands, such as sugars, proteins, peptides, or antibodies, can be used for active targeted drug delivery for the treatment of a variety of brain and CNS diseases [102].

For the treatment of GBM and other gliomas, one ligand that has been in the spotlight in recent years is transferrin [103]. TfR is expressed in brain capillary endothelial cells and has been shown to be highly overexpressed in astrocytoma cell lines, including in GBM. This means that Tf can be used to functionalize nanoparticles for enhancing their permeability across the BBB and for selectively targeting tumour cells [104]. A variety of actively targeted drug-delivery systems functionalized with Tf has been shown to possess enhanced BBB permeating properties in a number of studies [105]. A drug-delivery system that can readily cross the BBB and selectively target tumour cells is a massive leap towards enhancing the effectiveness of GBM chemotherapy.

#### 3.5.3. Surface Modification through Physical Adsorption Using Electrostatic Forces

Another way nanoparticles can be functionalized is by adsorption or ‘coating’ through surface modifications using surfactants (or other biomolecules) [106,107]. Some drug-loaded NPs coated with surfactants have also been shown to be more adept at crossing the BBB [107]. Although the mechanism through which many of the surfactants enhance BBB permeability is not fully known, it is theorized that surfactant-coated nanoparticles behave in a comparable manner to certain lipoproteins able to cross through the BBB via RMT [71].

## 4. Nanotechnologies for Brain Drug Delivery

Multiple modalities employing principles of nanotechnology have risen in popularity for uses in drug-delivery applications over the last few decades. Drug-delivery systems based on nanotechnologies come in a variety of types, such as inorganic systems, including gold nanoparticles (AuNPs), magnetic nanoparticles (MNPs), carbon-based nanoparticles, or, among others, transition metal dichalcogenide (TMDC) 2D nanomaterials [108,109]. Some organic delivery systems include polymeric micelles, dendrimers, solid lipid nanoparticles (SLPs) and polymeric solid nanoparticles (SNPs) [14]. Each of these delivery systems presents with its own specific set of advantages and limitations. An assortment of nano-delivery systems is currently being investigated for use in brain drug-delivery applications for the treatment of cancers. Positive data have been published in various studies exploring nanotechnological drug-delivery systems in cancer therapeutics. These studies have researched the passive as well as active targeting of drugs [70,110,111,112].

The passive targeting of a drug-loaded nanocarrier relies on the carrier’s ability to easily permeate tumour tissue and on their physiological stability. The amplified stability guarantees a longer half-life of the carriers in the bloodstream, prompting a higher concentration of the drug in the circulatory system for a longer duration [113]. The most significant feature of passive targeting is the size of the nanocarrier, as size is often the deciding factor between elimination and circulation [112]. Nanocarriers of appropriate size exploit the unique leaky vasculature of tumour cells and their weakened lymphatic drainage to sneak into the tumour microenvironment. As a result, nanocarriers (in the absence of any targeting group, i.e., passively targeted) can effectively localize in the microenvironment of a tumour due to their enhanced permeability and retention (EPR) effect [70,112,113]. However, recent studies over the last decade have concluded that the EPR hypothesis is not as universal or important as previously thought. Multiple recent studies have shown that the EPR effect works in in vivo models but is generally absent in humans [114,115,116]. While effective, passive targeting is often insufficient for treating cancers. There are a few constraints when using passive drug targeting, many of which arise from the inability of non-target cells to regulate carrier uptake. This often results in off-target accumulation of the drug. Off-target delivery of chemotherapy drugs may lead to the development of multi-drug resistance in the cancer cells, making them yet more difficult to kill, and is also the source of many of the adverse side effects patients experience during chemotherapy [117]. The inconsistency in the range of the EPR effect that different tumour cells can possess is a major limitation in passive targeting. This can result in decreased permeation of the drug carriers into the cellular microenvironment [110,118].

The limitations of passively targeted nanocarriers can be addressed using active targeting. In contrast to passive drug targeting, this form of drug delivery incorporates ligands that show a higher affinity towards the changed physiology of tumour cells [118]. Where passive targeting leads to an efficient localization of the nanocarriers in tumour microenvironments due to the EPR effect, carriers functionalized for active targeting promote selective uptake of the nanocarriers by the cancer cells themselves [112,119]. Ligands used for functionalizing nanocarriers for active drug targeting can range anywhere from small proteins or peptides to carbohydrates or polysaccharides, to other organic molecules, such as folate, aptamers, or hyaluronic acid [12,119].

### 4.1. Inorganic Nanoparticles

Inorganic nanoparticulate drug-delivery systems have been heavily investigated in the last two decades due to their unique physico-chemical characteristics, versatile and simple preparation techniques, (relatively) easy surface-functionalization and high biocompatibility. In addition to their usage in drug-delivery applications, inorganic nanoparticles (NPs) are also being used in theranostics, such as for photodynamic therapy (PDT) for cancer treatment [108]. Theranostics refer to personalized medicine. It involves targeted therapy based on specifically targeted diagnostic tests. Precision imaging and subsequent targeting requires the delivery of the theranostic cargo to the cancer-specific sites and a number of inorganic NPs are highly efficient in doing so owing to their size, biocompatibility and the versatility with which they can be decorated to target specific receptors or antigens [120,121,122].

Nanocarriers that actively target cancer cells are particularly promising in the treatment of brain and CNS diseases [36]. There are a variety of receptors that are abundant in the BBB, such as transferrin receptors, insulin receptors, low-density lipoprotein receptors and, among others, leptin receptors, that can be targeted by using ligands specific to the receptors to transport drugs across the BBB through receptor-mediated transcytosis [18,30].

#### 4.1.1. Gold Nanoparticles (AuNPs)

Gold NPs (AuNPs) make up a significant portion of all research in biomedical nanotechnological platforms since the field’s inception some decades ago. AuNPs possess unique chemical, physical, electrical, optical and biochemical properties which makes them highly useful in theranostic medicine [123,124]. Among their several biomedical applications, AuNPs’ high potential for utilization in targeted drug-delivery applications is the main facet that is focused on in this review [125,126].

**Figure 4:**
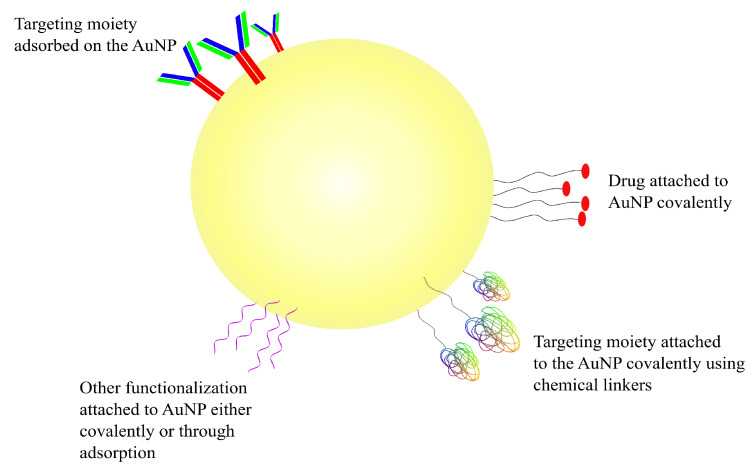
Schematic representation of a gold nanoparticle. Drugs and other functionalizations can be adsorbed or covalently attached onto AuNPs’ negatively charged surface.

AuNPs are very attractive vehicles to be used in drug-delivery applications due to their size, biocompatibility and their in vivo as well as ex vivo stability [124]. Depending on the method of preparation, AuNPs can be obtained in a range of sizes, from as small as 1 nm, increasing to larger than 100 nm, and of multiple shapes, such as spherical, rod-like and cubic, among others [127]. More importantly, the ability to functionalize gold NPs in a variety of ways is what differentiates them from other nanotechnological modalities used for drug delivery [125,128]. AuNPs have a negatively charged surface which enables them to be functionalized using a number of different biomolecules, such as DNA, peptides, proteins, or antibodies. The shape and the size of the nanoparticle determines its electrical properties; depending on the electric properties, AuNPs can be functionalized in one of two ways [129,130]. The first one is through covalent interaction between the surface of the NP and the functionalizing moiety, the result of which is a strongly bonded and stable complex; this is most commonly achieved through a sulphur-containing functional group such as thiol [123,129,130]. The second way through which AuNPs can be functionalized is through non-covalently adsorption of the decorator using electrostatic interactions, hydrophobic entrapment and/or van der Waals forces [131,132]. The covalent linkage of the drug or another substrate on AuNPs (or any other NPs, for that matter) requires some chemical modification of either the NP or the molecule which may impact the effectiveness of the ligand in undesirable ways; physical adsorption for facile decoration of the nanoparticle is one of the easiest ways around such an issue [131,132]. The different ways in which AuNPs can be targeted and/or functionalized can be seen in the schematic diagram represented in Figure 4.

While AuNPs present with a number of properties which can be highly useful in overcoming a number of challenges in drug delivery, they are not without their own set of flaws. Contradicting data have been published regarding the size-dependant toxicity of functionalized gold nanoparticles in a number of studies. Historically, their shape and size have been shown to influence toxicity to varying degree in a number of publications [133,134,135]. An example of AuNPs being cytotoxic can be seen in the publication by Gao et al. Their study showed higher in vitro cytotoxicity of AuNPs with a diameter of 8 nm than larger, 37 nm nanoparticles [136]. Contradictory results were observed by Rosli et al. who found their 50 nm AuNPs to be more cytotoxic than 13 nm and 70 nm nanoparticles in in vitro studies [137]. There are inconsistent data regarding the cytotoxicity of AuNPs and, until a universally accepted methodology to determine the profiles is developed, the clinical applications of AuNPs remain limited.

Various studies have shown AuNPs to not only be capable but also efficient in being able to cross the BBB. In their 2012 study, Prades et al. presented AuNPs conjugated to the β-sheet breaker peptide LPFFD, modified with a cystine (C) residue at the N-terminus (CLPFFD) (AuNP-CLPFFD) incorporated with THRPPMWSPVWP (THR)–a peptide that has been shown to target TfR—to have enhanced BBB permeation in in vitro and in vivo models for the treatment of Alzheimer’s disease [138]. The THR–AuNP–CLPFFD NP complex is theorized to cross the BBB via RMT, on account of THR being able to interact with TfR, which is abundant in the endothelial cells of the BBB [60].

In a study published in 2015, Sela et al. showed spontaneous accumulation of small-diameter AuNPs in the BBB in mice and proposed another transport route that can be exploited to enhance the BBB permeation of AuNPs. Their in vivo models suggested that potassium (K${}^{+}$), sodium (Na${}^{+}$) and calcium (Ca${}^{2+}$) ion channels directly affected the BBB permeation of AuNPs. The researchers’ findings suggested the transport of small AuNPs across the BBB through the tight junctions between the endothelial cells as opposed to the transcytotic route proposed by Prades et al. [139].

Other publications have also demonstrated the potential for using AuNPs for the treatment of brain and CNS disorders. For example, functionalized AuNPs for the treatment of Alzheimer’s and Parkinson’s diseases were presented in studies published in 2017 and 2019, respectively, with promising results [140,141].

#### 4.1.2. Magnetic Nanoparticles (MNPs)

**Figure 5:**
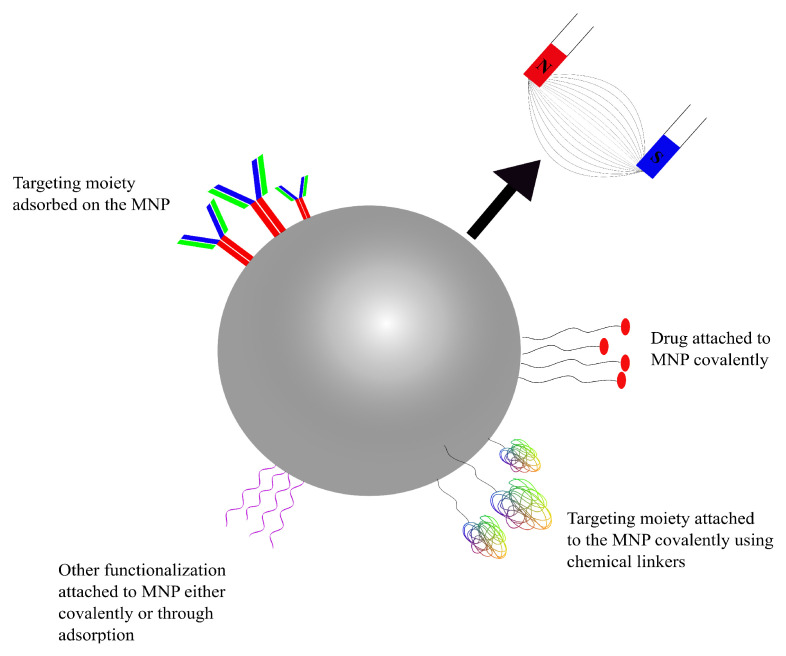
A schematic representation of a functionalized, drug-carrying magnetic NP. Targeting moieties and drugs can either be adsorbed or covalently attached using linkers.

Using magnetic nanoparticles (MNPs) in drug-delivery applications was first introduced in the late 1970s [142]. Therapeutic agents can be entrapped into or attached to the surface of magnetic nanoparticles, before administration into the bloodstream. MNPs can be targeted using conventional active-targeting techniques (using RMT or AMT) or through a magnetic field applied externally focusing on the target site, which can then localize the NPs at the desired site [143,144]. MNPs can be used as drug-delivery systems with a greater control on drug release and can be used for diagnostic purposes through visualization using magnetic resonance imaging (MRI). As MNPs can rely on an external magnetic force to drive them to the therapeutic target, the control over where the nanoparticles are to be localized is diminished with the increase in depth (distance) within the body. As such, there have only been few clinical trials for MNPs [145]. Where the delivery mechanisms of the previously discussed drug-delivery systems were dictated by the biochemistry of the BBB, MNPs targeted using an external magnetic field are different in that they can be ‘dragged’ across the BBB using said magnetic force (Figure 5) [146].

A number of different types of MNPs have been studied and many of them have resulted in conclusions worthy of further studies [146,147,148]. Chertok et al. showed effective targeting or brain tumours using magnetic iron oxide (IO) nanoparticles in at least three different study publications [149,150,151]. A 2012 study developed Lf-conjugated PEGylated (conjugation with the polymer poly(ethylene)glycol; more details in Section 5.3.1) IO NPs that were able to cross the BBB using RMT in in vitro and in vivo models [143]. Tf-conjugated, *fluorescein isothiocyanate isomer I* (FITC)-loaded IO nanoparticles (Tf-FTIC-IO MNPs) were shown to cross the BBB without disrupting it, using an in vivo model in a 2013 study. The Tf–FTIC–IO MNPs permeated the BBB and diffused into the brain through Tf-mediated RMT [152].

### 4.2. Organic Nanoparticles

#### 4.2.1. Polymeric Micelles (PM)

**Figure 6:**
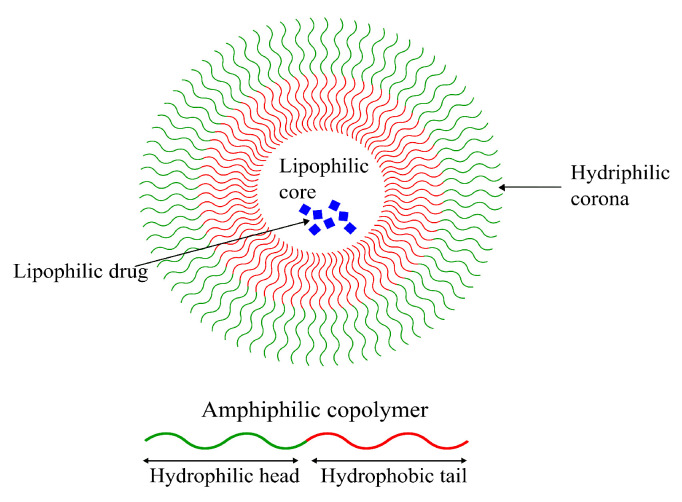
Schematic representation of a polymeric micelle formed by an amphiphilic polymer with a lipophilic drug encapsulated in its lipophilic core. Hydrophilic drugs can also be encapsulated in polymeric micelles due to their amphiphilic nature.

Formed from amphiphilic polymers, micelles are characterized by their unique spherical shape, resulting from the hydrophobic section(s) segregating from the hydrophilic section(s), as seen in Figure 6. This leads to the formation of an inner hydrophobic core surrounded by external hydrophilic terminals [153]. Their amphiphilicity enables them to be stable in physiological environments, giving them long circulation times and providing them with sufficient time to reach their target tissue [154]. Despite the long systemic circulation, concerns regarding their poor cellular binding and uptake are still present [155]. Due to their amphiphilic nature, lipophilic as well as hydrophilic drugs can be loaded into micellar DDSs, making them highly versatile vehicles for chemotherapeutics. Despite that, micellar drug-delivery systems possess low drug loading capacities compared to other systems (discussed in the next sections) [155,156,157].

Micellar DDSs are popular owing to their small size, long circulation time, good stability and targetability (functionalizability) [154]. Sezgin-Bayindir et al. extensively studied micelles formed using a number of block copolymers, focusing specifically on their ability to function as drug carriers to treat brain and CNS diseases. They studied micelles formed from block copolymers *poly(styrene)-poly(acrylic acid)* (PS-PAA), *poly(ethylene glycol)-b-poly(lactic acid)* (PEG-PLA) and *distearyl-sn-glycero-3-phosphoethanolamine-N-methoxy poly (ethylene glycol)* (PEG-DSPE). The study concluded that only micelles formed from the copolymer PEG${}_{5000}$-PLA${}_{4500}$ were worthy of further considerations for usage in brain drug delivery [158].

Another study published in 2018 by Abourehab et al. showed that the drug Dapoxetine (DPX) loaded in polymeric micelles formed from the PEG-PLGA block copolymer had a higher bioavailability in the brain compared to just the drug alone. They concluded a 2.7-fold increase in DPX-content in the brain using DPX–PEG–PLGA micelles compared to DPX commercial tablets following oral administration using in vivo models [159]. A 2008 study showed that Ciprofloxacin—an antibiotic—was able to readily cross the BBB when encapsulated in micelles formed using cholesterol-conjugated PEG, decorated with transcriptional activator TAT peptide using in vivo models [160].

#### 4.2.2. Dendrimers

**Figure 7:**
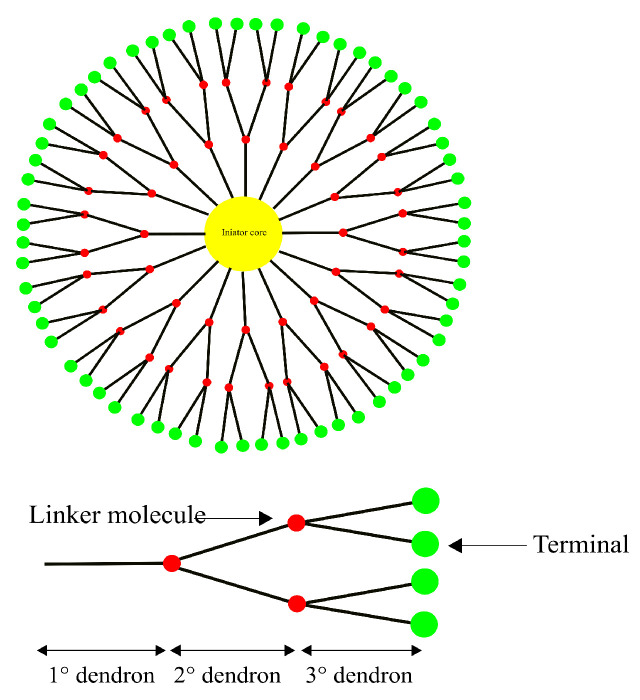
A generic structure of a 3rd generation dendrimer consisting of a core, dendrons and the terminal group. Drugs and functional moieties are typically loaded onto the terminal group.

Dendrimers are highly branched spherical polymers. They have become popular in the last couple of decades as drug-delivery systems due to their size and the relative ease with which they can be synthesized and modified compared to other nanotechnology-based drug-delivery systems. Due to their branching, dendrimers are sometimes referred to as “starburst” polymers. They are composed of three main, distinct architectural components, i.e., (1) an initiator core, to which (2) interior layers (generations) of repeating subunits (dendrons) are radially linked, and (3) terminals, which is where the majority of the functionalization as well as drug loading takes place [161,162]. A schematic representation of a generic dendrimer can be seen in Figure 7. The linking of bioconjugates such as proteins or antibodies onto the dendrimer surface has also been seen as an attractive feature due to their extensive branching [156,163].

**Figure 8:**
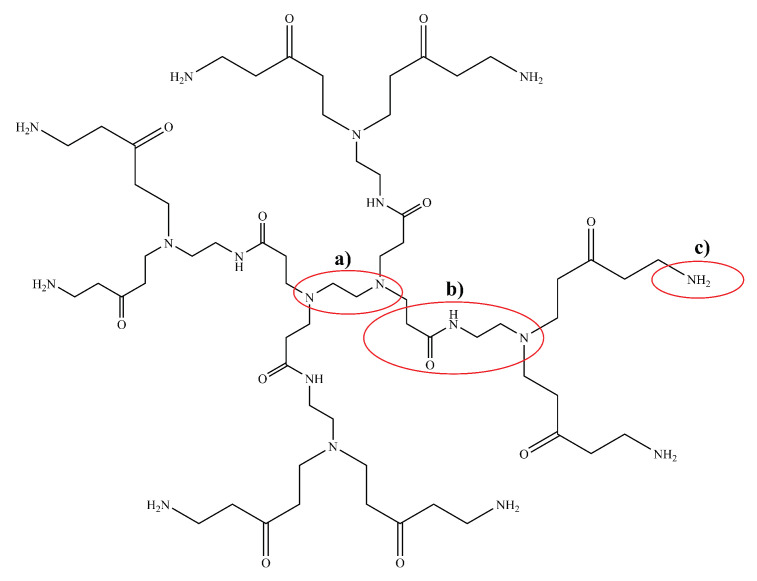
Chemical structure of a 1st generation poly(amidoamine) (PAMAM) dendrimer with (**a**) an ethylene diamine core, (**b**) amidoamine dendrons and (**c**) an amino terminal group. There are a number of different terminal groups that PAMAM dendrimers are available in, making them very versatile for drug-delivery applications.

*Polyamidoamine* (PAMAM) dendrimers (Figure 8) are one of the most studied classes of dendrimers used in the delivery of therapeutics to the brain. They have an ethylenediamine (C${}_{2}$H${}_{4}$(NH${}_{2}$)${}_{2}$) core, amide (RC(=O)NR${}^{\prime }$R${}^{\prime \prime }$) branches (where R, R${}^{\prime }$ and R${}^{\prime \prime }$ are organic groups or hydrogen atoms) forming the walls of cavities and amino (-NH${}_{2}$), hydroxyl (-OH), or carboxylic acid (-COOH) functional groups as terminals [164,165]. The amino-terminated variants of PAMAM are the most popular in pharmaceutical research owing to their ease of bioconjugation using a variety of different protein and/or peptide ligands. The amide dendrons of PAMAM dendrimers are similar to the peptide backbones of proteins. PAMAM dendrimers are small, highly stable, highly water-soluble and easily biofunctionalizable, making them very suitable for the neuro-delivery of therapeutics [166]. The presence of cavities and the amino terminals of PAMAM dendrimers means that drugs can either be conjugated using chemical linkage or they can be encapsulated within the cavities [164]. Dendrimers can vary significantly in size and other physical characteristics; therefore, drug-delivery systems based on them can use any among of transcellular passive diffusion, paracellular transport, CMT, RMT, or AMT to travel across the blood–brain barrier [165].

PEGylated-PAMA dendrimers decorated with glioma-honing peptide (Pep-1) (PEP1-PEG-PAMAM) have been shown to possess enhanced BBB permeability profiles in in vitro and in vivo models. This complex is theorized to be passing through via RMT, exploiting endocytosis by *interleukin-13-receptor-α2* (IL-13Rα2) [167]. Another study showed PEG-PAMAM dendrimers to be effectively used to deliver drugs to the brain for ischemic stroke therapy [168].

#### 4.2.3. Liposomes

**Figure 9:**
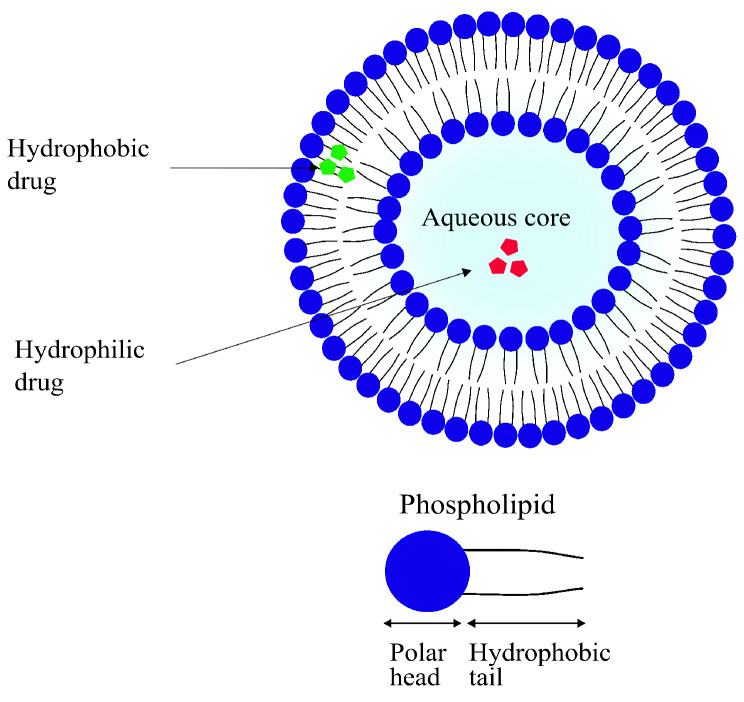
A schematic representation of a simple liposome with an aqueous core and phospholipid bilayer. Hydrophilic (red) as well as lipophilic drugs (green) can be encapsulated in liposomes.

Liposomes are small, spherical vesicles composed of one or more concentric spheres of phospholipid bilayers separated by aqueous compartments, as seen in Figure 9. They are amphiphilic in nature, meaning they have a hydrophilic core and a hydrophobic (lipophilic) tail. Most of their physical properties such as surface charge, size and amphiphilicity can be modified depending on the method of preparation and the choice and quantity of the lipid used. Their sizes can range anywhere from 50 nm to 1 μm [169,170]. As the main component of liposomes is the phospholipid bilayer, they are highly biocompatible. They are often regarded as the first generation of DDSs with their first reported use in delivery applications dating back as far as 1971 [171,172].

Controlled and targeted delivery of therapeutics using liposomes has several advantages over some other modalities that are discussed later in this paper. For example, as previously discussed, they are highly biocompatible. Due to their biphasic (lipid and aqueous compartments) nature, liposomes can be used to carry lipophilic as well as hydrophilic drugs efficiently. Lastly, liposomes can be functionalized in several ways for the targeted delivery of drugs [169,170]. Some of the disadvantages that come with using liposomal DDSs include poor solubility, leading to shorter circulation times. Their comparatively quick degradation has also been reported to be a significant cause of premature leakage of the encapsulated drug. On top of all that, the production of liposomal drug-delivery systems is costly, significantly limiting their overall potential of application [117,173].

Traditional techniques used for liposomal production have generally utilized the thin-film hydration technique. The generic workflow involves formation of a thin film of the phospholipid through dissolution in an organic solvent and subsequent evaporation using a rotary evaporator, resulting in thin lipid film which is then hydrated with large volumes of an aqueous solution [174]. Other commonly used techniques to prepare liposomes include reverse-phase evaporation, detergent dialysis and solvent injection [175,176,177]. The major limitations surrounding these techniques include the lack of control (therefore, lack of reproducibility) of particle size; poor stability of the prepared vesicles, because of residual organic solvent; and extreme difficulty in sterilizing the liposomes for clinical use (as many lipids are heat-sensitive) [178].

A number of liposomal drug-delivery systems has been effectively used for brain drug delivery. For example, in their 2017 publication, Gurturk et al. prepared DSPE-PEG liposomes decorated with *maltodextrin* for the delivery of Levodopa for the treatment of Parkinson’s disease. The researchers obtained liposomes with low polydispersity and hydrodynamic diameters between 100 and 150 nm, with decent drug loading. In vitro studies showed a statistically significant higher amount of Levodopa when encapsulated into maltodextrin-conjugated DSPE-PEG liposomes compared to non-targeted liposomes and the drug alone. Maltodextrin is known to cross the BBB via RMT [179].

Another study published in 2019 showed significantly improved delivery of *pituitary adenylate cyclase-activating polypeptide* (PCAP) to the brain when encapsulated in DSPE-PEG liposomes conjugated with *gH625*—a peptide derived from *Herpes simplex virus 1* using an in vitro rat model [180]. The gH625 peptide has been shown to be efficiently internalized by neuroblastoma and astrocytoma cells in previously published in vivo studies, suggesting this could be an efficient functionalization for future brain drug-delivery applications [181].

Other liposomal systems for brain drug delivery have also been studied. Recent advancements have been summarized in previous publications [182,183].

#### 4.2.4. Niosomes

**Figure 10:**
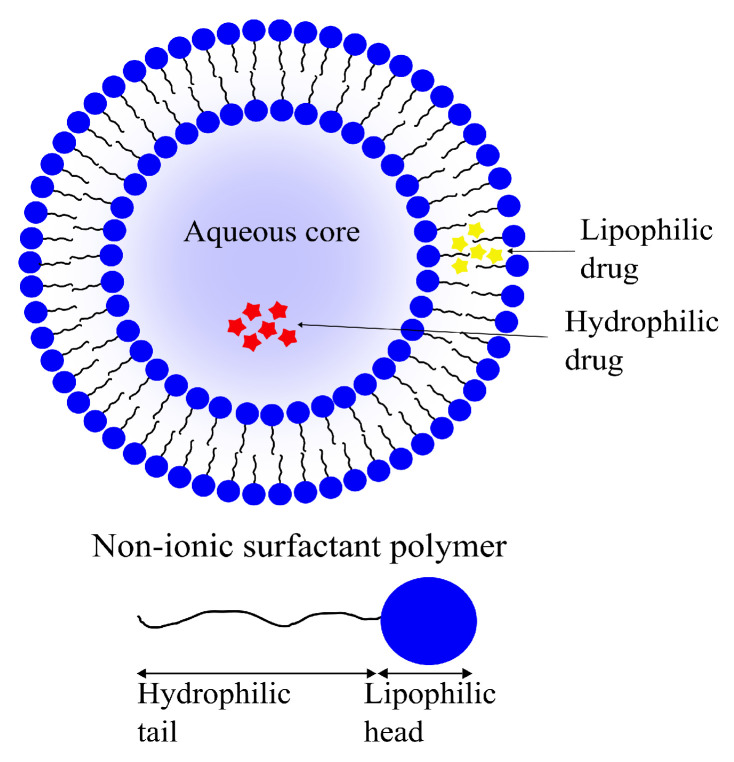
A schematic representation of a niosome formed with amphiphilic, non-ionic surfactants with an aqueous core. Similar to liposomes, lipophilic (yellow) as well as hydrophilic drugs (red) can be encapsulated in niosomes.

Niosomes, another class of vesicular DDSs, are self-assembling vesicles formed by amphiphilic non-ionic surfactants in aqueous environments. Niosomes are analogous to liposomes structurally as well as functionally (Figure 10). Similar to the phospholipid vesicles, niosomes can be used as drug carriers for hydrophilic as well as lipophilic molecules due to their amphiphilicity. Niosomes have the added bonus of being more cost-effective to produce and stable than their phospholipid-based cousins [184,185].

Niosomes can be prepared using various techniques dictated by the desired physical properties, such as size and diameter, choice of drug to be entrapped and the number of double layers in the vesicle. A common niosome preparation technique involves probe sonication of a drug-containing aqueous phase following addition into a surfactant solution. Other techniques used for their preparation include thin-film hydration and some others, the details of which extend beyond the scope of this review, such as micro-fluidization and, among others, reverse-phase evaporation [186,187].

Only a small number of niosomal brain delivery systems have been reported in recent years. In their 2018 study, De et al. reported a 3.04-fold increase in the concentration of temozolomide when encapsulated in niosomes decorated with the peptide *chlorotoxin* for the treatment of GBMs. The resulting niosomes had a reported diameter of 220 nm and had a high drug loading capacity of 79% [188].

#### 4.2.5. Microemulsions

**Figure 11:**
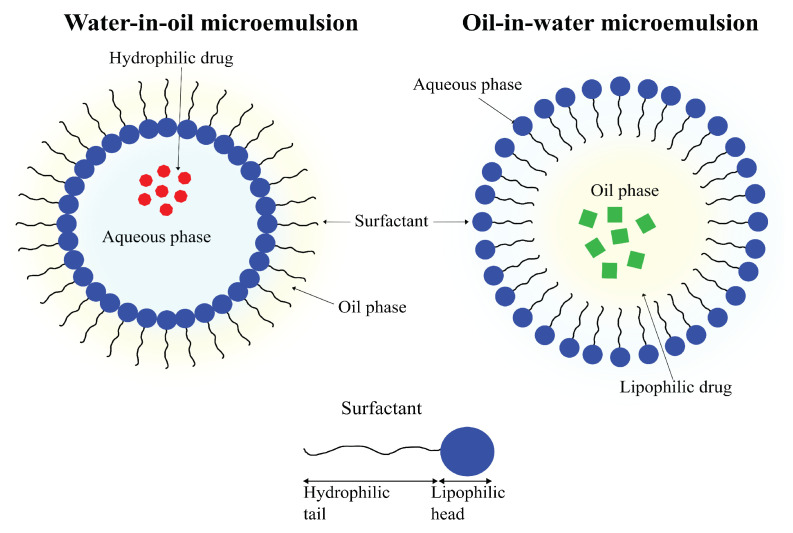
A schematic representation of a water-in-oil microemulsion droplet with a hydrophilic drug entrapped in its aqueous core (left) and an oil-in-water microemulsion droplet with a lipophilic drug encapsulated in the organic core.

An emulsion refers to a mixture of two (or more) immiscible liquids. As the name suggests, microemulsions are emulsions on a micro-scale. Where emulsions are thermodynamically unstable, with phase separation being an inevitability, microemulsions are thermodynamically stable and can remain in a diphasic stage indefinitely. Another difference between the two is in the appearances; emulsions tend to be milky, whereas microemulsions are usually clear. Microemulsions are defined as mixtures of oil, water and surfactant having droplet sizes of colloidal dimensions, frequently with droplet diameters of less than 100 nm (Figure 11) [189,190,191].

Microemulsions are easily prepared and can be used to carry lipophilic as well as hydrophilic drugs. Their small droplet size and high thermodynamic stability enables microemulsions to possess solubilization properties that make them highly attractive tools for use in drug-delivery applications [191].

Similar to niosomes, only a small number of studies report brain drug delivery using microemulsions. A 2014 study by Patel et al. reported the preparation of drug-loaded microemulsions for intranasal delivery of Carbamazepine to the brain. The prepared formulations were reported to be stable for up to 6 months under standard conditions. The concentration of the drug Carbamazepine was found to be significantly higher compared to IV administration using in vivo models [192].

#### 4.2.6. Polymeric Solid Nanoparticles (SNPs)

These are solid, colloidal particles formed by polymers. Depending on the type of nanoparticles, drugs can be dissolved, adsorbed, or encapsulated. Polymeric NPs are highly modifiable and can exhibit a broad range of physico-chemical and biochemical characteristics. Like other types of solid nanoparticulate DDSs, SNP (as many other types of nanoparticles) delivery systems can be used for the active or passive targeting of drugs to a range of tissues because of their high functionalizability [156]. The benefits, constraints, methods of preparation and kinds of polymeric nanoparticulate drug carrier systems are outlined in more detail in the following section.

## 5. SNPs in Drug-Delivery Applications

A number of easily accessible and biologically safe polymers has been introduced for use in drug-delivery systems over the past two decades. Many polymers, such as *poly(lactic-co-glycolic acid* (PLGA) and PEG, have risen to the occasion due to their biocompatible nature.

### 5.1. Functionalization of SNPs

**Figure 12:**
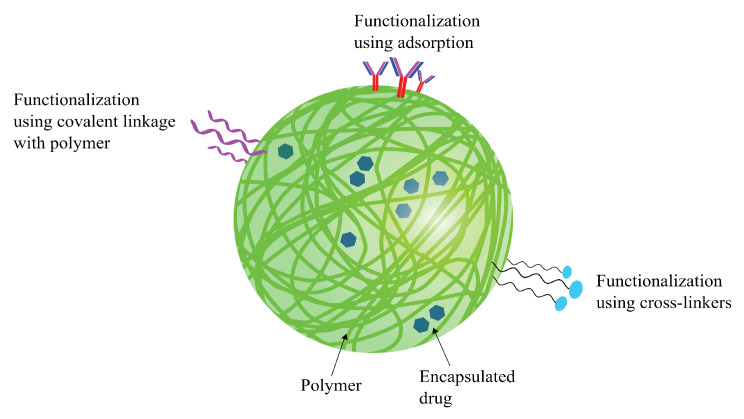
A schematic representation of an SNP which can be functionalized using covalent linkage through crosslinkers as well as through adsorption.

SNPs can be functionalized in several different ways, as discussed in Section 3. Solid polymeric nanoparticles coated with surfactants such as tween-80 or poloxamer-188 are known to adsorb plasma proteins such as Apolipoprotein A1 (Apo-A1) or E (Apo-E) onto their surface, which enables them to cross the BBB via RMT [193,194]. Other than surfactants, certain polysaccharides adsorbed onto SNPs made from polymers such as polylactic acid (PLA) and PLGA have been shown to be some of the most biocompatible DDSs [100]. Biomolecules such as proteins on SNP surfaces have also shown to impact the way the drug carrier behaves in the body. Many protein-adsorbed SNPs have increased cellular uptake due to specific binding of the proteins with their respective receptors expressed on the cell membrane [195,196]. The common ways used to functionalize SNPs for drug-delivery applications is summarized in Figure 12.

#### SNPs Made Using Layer-by-Layer (LbL) Assembly Method

Hollow, multilayered capsules that can be used for drug delivery can be made using a technique referred to as ‘layer-by-layer’ (LbL) assembly. These NPs possess properties, which are discussed in detail in the following section, which have prompted extensive research into this technique. For example, these are highly functionalizable nanoparticles and a range of chemicals can be encapsulated into them with an impressive carrying capacity. The “stepwise” nature of LbL assembly allows a wide range of functionalizations to be incorporated within the nanoparticles instead of surface or end-group modifications. Another reason such multilayered NPs have garnered attention is due to the sustained drug-release, which can be easily controlled through environmental conditions such as pH or an external magnetic field [197,198,199].

### 5.2. Types of SNPs: Nanocapsules and Nanospheres

**Figure 13:**
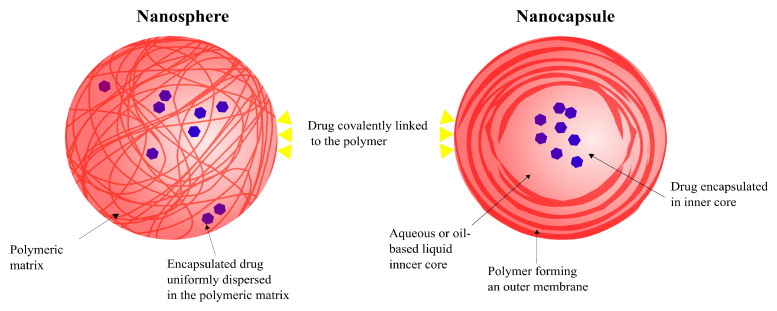
Schematic representation highlighting the differences between the two types of solid polymeric nanoparticles, nanospheres and nanocapsules.

The use of polymeric nanoparticles for drug delivery has several advantages over other systems, such as the ability to control and manipulate the release of the drug, which not only can diminish the intensity and number of adverse side-effects but can also increase the efficacy of the drug. This system can be used to deliver drugs via a number of administrative routes such as oral, nasal, parenteral, etc. [63,75]. There are two main types of polymeric nanoparticles that are being studied for applications in drug delivery, nanocapsules and nanospheres (Figure 13) [75].

Nanospheres are solid, spherical nanoparticles whose polymeric chains form a matrix, into or onto which the drug can be dissolved, entrapped, encapsulated, or attached. The drug is uniformly dispersed into the matrix of these nanoparticles [200,201]. In comparison, nanocapsules are vesicular particles with a liquid inner core (which can either be aqueous or oil-based) and a polymeric outer membrane. The drug is normally loaded in nanocapsules via encapsulation into the liquid core but can also be attached onto the particles’ surface through covalent linkage [202]. The type of polymer and the technique used to prepare the nanoparticles are the two main factors that determine whether the resulting carrier system is a nanosphere or a nanocapsule [67,203]. While there are no universally accepted conclusions about size differences between nanospheres and nanocapsules, a study by Teixeira et al. found PLGA nanospheres to be smaller (170 nm) than PLGA nanocapsules (300 nm) [203].

Depending on the drug’s physico-chemical properties and the respective drug-release mechanisms of nanosphere and nanocapsule drug carriers, drug release can differ to a varying degree between the two systems [204]. In nanospheres, the loaded drug is released following the degradation of the matrix. A controlled drug release when using nanosphere carriers is achieved by controlling the rate of matrix degradation. The release of drugs from nanocapsules is diffusion-controlled—i.e., the release is dependant on cross-membrane concentration, that is, the concentration gradient between the inner compartment of the nanoparticle and the bulk environment in which it is located—or burst release—i.e., a large amount of drug is immediately released after being introduced into the release medium before achieving stability [205,206]. Therefore, the type of polymeric nanoparticle system for drug delivery is chosen based on factors such as the physico-chemical properties of the drug, the physiological conditions of the target tissue/cells and the circulation life (physiological stability and elimination) of the nanoparticle [69,204].

### 5.3. Commonly Used Polymers for SNPs in Brain Drug Delivery

#### 5.3.1. PLGA and PEG

**Figure 14:**
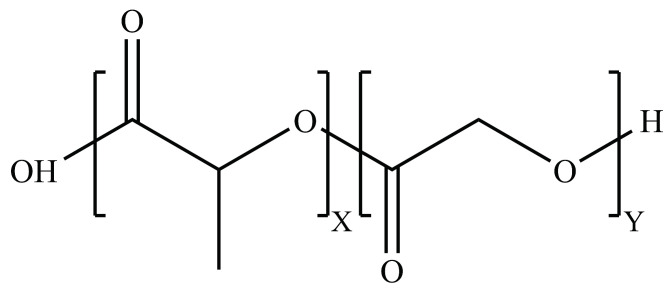
Chemical structure of the copolymer *poly(lactic)-co-(glycolic) acid* (PLGA), which is widely used in drug-delivery applications. X represents the number of units of lactic acid and Y represents the number of units of glycolic acid.

**Figure 15:**
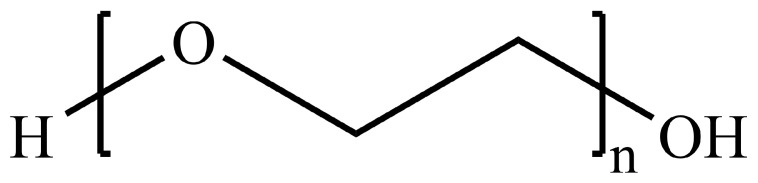
Chemical structure of the non-ionic polymer, poly(ethylene)glycol (PEG). PEG is also widely used in nanomedicinal applications. The n represents the repeating subunits of ethylene glycol.

Some polymers, such as PLGA (Figure 14) and PEG (Figure 15), have displayed high potential to be used for applications in targeted drug delivery to the brain. PLGA nanoparticles are being widely looked at as a possible nanocarriers for drugs to enable passage through the BBB owing to their high biocompatibility, biodegradability and functionalizability [65,207]. PLGA hydrolyzes into lactic acid (LA) and glycolic acid (GA) in aqueous environments, both of which are easily metabolized and eliminated from the body through the Krebs cycle [207]. While PLGA SNPs do not have a sufficiently long half-life after IV administration on their own, this can be significantly prolonged by exploiting the polymer’s highly modifiable end-group [207,208]. PLGA, on its own, has a negative surface charge that causes it to have a poor uptake by cells; therefore, it is not able to readily cross the BBB. These drawbacks can be mitigated through the use of crosslinkers, surface adsorption and end-group modifications [63,207].

A study published by Barbara et al. in 2017 showed PLGA SNPs loaded with curcumin (Cur) decorated with the glycopeptide “g7” (Gly-l-Phe-D-Thr-Gly-l-Phe-l-Leu-LSer(O-β-D-Glucose)-CONH${}_{2}$) [g7-(Cur-PLGA-Cur)], which were reported to have significantly reduced β-amyloid aggregation, which is a significant indicator of Alzheimer’s disease [209]. Another study, published in 2010, showed increased accumulation of trimethylated chitosan (TMC)-conjugated PLGA NPs (TMC-PLGA NPs) using an in vivo model. These nanoparticles exhibited negligible cytotoxicity and were shown to have been extensively distributed in the periventricular region of the cortex and the third ventricle of the brain. The researchers believe AMT to be the driving force behind TMC-PLGA NPs’ ability to cross the BBB [210].

PEG is another highly modifiable polymer that is being studied for drug-delivery applications. Similar to PLGA, it exhibits low toxicity levels in the body, is biocompatible and, while not biodegradable, is easily eliminated from the body by the kidneys. PEG has, as a result, evolved into a commonly used polymer in a number of pharmaceutical applications, including nanomedicine [65]. PEGylation—covalently linking PEG onto another molecule (another polymer, NP, drug, protein, antibody, etc.)—is a common technique used for the functionalization of a number of nanoparticulate drug carriers. Some PEGylated drugs have, in fact, been approved and are currently in use. For example, PEGylated liposomal Doxorubicin is a Health Canada and Food and Drug Administration (FDA)-approved nanomedicine sold under the brand names *DOXIL^®^* (in the US) and *Caelyx^®^* (in the rest of the world) by Tibotec Therapeutics—a division of Ortho Biotech Products in New Jersey, USA [211]. DOXIL/Caelyx^®^ is primarily being used in the treatment of ovarian cancer, breast cancer, multiple myeloma and Kaposi’s sarcoma [212]. The PEGylation of drug-loaded PLGA nanoparticles (PEG-PLGA NPs) has shown to be an effective technique for surface modification to PLGA NPs for brain drug delivery, with the PEGylated nanoparticles possessing noticeably improved BBB permeating properties [65,213].

#### 5.3.2. PLGA–PEG–PLGA (PEP)

**Figure 16:**
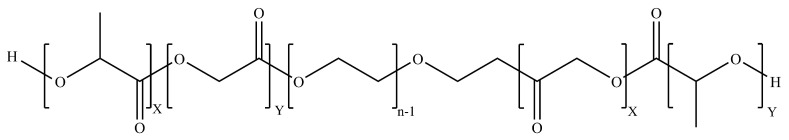
Chemical structure of the multiblock copolymer PLGA–PEG–PLGA (PEP). The X and Y represent the numbers of repeating subunits of lactic and glycolic acid, respectively, and the n represents the number of ethylene glycol subunits. Nanoparticles made using PEP have been shown to possess characteristics that encourage further research into their usage for brain drug-delivery applications.

Polymers made from more than one single type of monomer are referred to as copolymers. The PLGA–PEG–PLGA (PEP) triblock copolymer (Figure 16), first synthesized by Jeong et al., has exhibited a considerable increase in the delivery of an encapsulated drug to the brain in a study conducted by Chen et al. [214,215]. The PEP copolymer can undergo further modifications to increase the amount of drug for the targeted and sustained delivery of chemotherapeutics to various targets within the body [215].

## 6. Discussion and Conclusions

Research in a wide array of fields is being conducted to determine the best mode of transporting therapeutics to the brain. Nanotechnology-based drug-delivery systems have been at the forefront of a large portion of such research studies. A variety of inorganic as well as organic NP-based drug-delivery systems are being researched for brain drug delivery. Each of these different classes of nanoparticles has its own set of advantages and drawbacks when it comes to drug-delivery applications, such as poor biocompatibility profiles, low drug loading capacities, or, among others, undesired physico-chemical characteristics. Solid, polymeric nanoparticles have especially been in the spotlight due to the sheer flexibility they provide. Depending on the type of polymer and method of production used, these nanoparticles can encapsulate a wide range of drugs; they can be modified to possess the desired physical, chemical and physiological properties to a degree far greater than a lot of the other types of nanoparticles.

The major hurdle that needs to be addressed for brain drug delivery is the BBB and there have been numerous published studies that show encouraging data. Whilst promising results have been seen for a number of these nanoparticles, there is still no reliable mode of transporting therapeutics to the brain. Therefore, further research must be conducted.

## Data Availability

All data available are reported in the article.

## Abbreviations

The following abbreviations are used in this manuscript:

DDS(s)	Drug-delivery system(s)
BBB	Blood–brain barrier
CNS	Central nervous system
CMT, RMT and AMT	Carrier-mediated, receptor-mediated and absorptive-mediated transcytosis
P-gp	P-glycoprotein
WHO	World Health Organization
GBM	Glioblastoma multiforme
Tf, oTf and Lf	serum-Transferrin, ovo-Transferrin and Lactoferrin
TfR	Transferrin receptor
EPR	Enhanced permeability and retention
NP(s)	Nanoparticle(s)
AuNP(s)	Gold nanoparticle(s)
MNP(s)	Magnetic nanoparticle(s)
MRI	Magnetic resonance imaging
PM(s)	Polymeric micelle(s)
SNP(s)	Polymeric solid nanoparticle(s)
Apo-A1 and Apo-E	Apolipoprotein A1 and Apolipoprotein E
LbL assembly	Layer-by-layer assembly
LA and GA	Lactic acid and Glycolic acid
**Abbreviated Chemicals:**
FTIC	Fluorescein isothiocyanate
IO	Iron oxide
PS-PAA	Poly(styrene)-poly(acrylic acid)
PEG-PLA	Poly(ethylene)glycol-poly(lactic acid)
PEG-DSPE	Distearyl-sn-glycero-3-phosphoethanolamine-N-methoxy poly
	(ethylene glycol)
PEG-PLA	Poly(ethylene glycol)-poly(lactic acid)
PEG-PLGA	Poly(ethylene glycol)-poly(lactic-co-glycolic acid)
DPX	Dapoxetine
PAMAM	Polyamidoamine
IL-13Rα2	Interleukin-13-receptor-α2
EDC	N-Ethyl-N${}^{\prime }$-(3-dimethylaminopropyl)carbodiimide hydrochloride
NHS	N-hydroxysuccinimide
PLGA	Poly(lactic-co-glycolic acid)
PEG	Poly(ethylene(glycol
BSA	Bovine serum albumin
FRα	Folate receptor alpha
Cur	Curcumin
g7	Gly-l-Phe-D-Thr-Gly-l-Phe-l-Leu-LSer(O-β-D-Glucose)-CONH${}_{2}$
TMC	Trimethylated chitosan
PEP	PLGA-PEG-PLGA
